# Role of microtubule-associated protein 6 glycosylated with Gal-(β-1,3)-GalNAc in Parkinson's disease

**DOI:** 10.18632/aging.102072

**Published:** 2019-07-09

**Authors:** Li Ma, Jiaxin Song, Xueying Sun, Wenyong Ding, Kaiyang Fan, Minghua Qi, Yuefei Xu, Wenli Zhang

**Affiliations:** 1 Department of Epidemiology, Dalian Medical University, Dalian 116044, China; 2 Biochemistry and Molecular Biology Department of College of Basic Medical Sciences, Dalian Medical University, Dalian 116044, China; 3 Medical Administration Department, Affiliated Hospital of Jining Medical University, Jining 272000, China

**Keywords:** microtubule-associated protein 6, Parkinson's disease, MPTP-induced mice, MAP6, glycosylation

## Abstract

Aberrant glycosylation of proteins has major implications for human diseases. To determine whether protein glycosylation contributes to the pathogenesis of Parkinson’s disease (PD), a mouse model of PD was established by injection of 1-methyl-4-phenyl-1,2,3,6-tetrahydropyridine (MPTP). Induction of PD-like features was verified by assessing motor impairment and confirming reductions in biological markers, including dopamine, 5-hydroxytryptamine and tyrosine hydroxylase, as well as the aggregation of α-synuclein. Altered glycosylation was detected using biotinylated agaracus bisporus lectin, which specifically binds exposed Gal-(β-1,3)-GalNAc linked to glycoproteins. Subsequent lectin affinity chromatography coupled with mass spectrometry revealed enhanced glycosylation of microtubule-associated protein 6 (MAP6) in PD mice as compared to healthy controls. In situ dual co-immunofluorescence analysis and immunoblotting confirmed that MAP6 is glycosylated with Gal-(β-1,3)-GalNAc oligosaccharides, which in turn alters the distribution and structure of MAP6 complexes within neurons. This is the first study to described MAP6 as a glycoprotein containing Gal-(β-1,3)-GalNAc oligosaccharides and to show that hyperglycosylation of MAP6 is strongly associated with the pathogenesis of PD. These findings provide potentially valuable information for developing new therapeutic targets for the treatment of PD as well as reliably prognostic biomarkers.

## INTRODUCTION

Parkinson’s disease (PD) is the second most common degenerative disorder of the central nervous system after Alzheimer’s disease, afflicting 1-2 % of the worldwide population older than 60 years [1]. Moreover, the incidence of PD is trending upward as the world’s population ages. Clinically, the majority of PD patients suffer from motor impairments involving resting tremor, bradykinesia, rigidity, and postural instability, along with non-motor symptoms that include dementia, olfactory dysfunction, sleep disturbance, depression, constipation, obsessive-compulsive behavior, and urinary difficulty [2, 3]. This morbidity caused by PD strongly correlates with an increased risk of death [3]. The most efficacious treatment for symptoms of PD is Levodopa [4–6]. However, its effectiveness declines with progression of the disease and is associated with numerous side effects, including dyskinesia, atherosclerosis, depression and dementia.

Breakthroughs shedding light on the genetic basis of PD have improved our understanding of the pathogenesis of PD and enabled identification of potential targets for neuroprotective therapies. However, the role of genetics in the etiology of PD is controversial. Although it is known that PD is a disorder of the extrapyramidal system and results primarily from degeneration of nigrostriatal neurons, the primary cause and neurological mechanism leading to PD has remained obscure [7–9]. Most likely, the cause is multifactorial and involves both environmental and genetic factors.

Post-translational protein modification is a mechanism by which protein functionality is regulated [10]. Glycosylation is a common modification in which an oligosaccharide is attached to a protein, thereby affecting its solubility, structure, charge or sensitivity to proteolysis, and thus functionality [11, 12]. Abnormal glycosylation has major implications for human health and is associated with such human diseases as congenital muscular dystrophy, IgA nephropathy, and rheumatoid arthritis, among others [13–18]. Glycosylation is found ubiquitously throughout the central nervous system [19], where protein or lipid-linked sugars play important roles in many developmental and functional processes, including cell-extracellular matrix and cell-cell interactions, neuronal migration, adhesion and axonal guidance [19]. In the context of neurodegenerative diseases, significantly altered glycomes have been noted in Huntington's disease, Alzheimer’s disease, and human Creutzfeldt-Jakob disease. Although little is currently know about the involvement of glycome changes in PD, it has been shown that α-synuclein contains O-linked sugars and its increased O-GlcNAcylation prevents protein aggregation that correlates with PD pathogenesis [12].

Our aim in the present study was to detect aberrantly glycosylated proteins in PD and establish the association between glycosylation changes and PD pathogenesis so as to further understanding of Parkinsonian risk factors, elucidate pathogenic mechanisms underlying PD, and identify promising biomarkers of PD prognosis and/or a potentially more effective drug targets for PD treatment.

## RESULTS

### Verification of the PD mice treated by MPTP

Neurotoxins such as 1-methyl-4-phenyl-1,2,3,6-tetrahydropyridine (MPTP), paraquat, and rotenone are widely used to reproduce the essential symptoms of PD in animal models. Among those models, MPTP-induced PD in mice is a well-established parkinsonian animal model that exhibits high symptomatic similarity to human patients with PD. In the present study, we confirmed the pathogenic effects of MPTP on the biochemical and motor function of the treated mice.

### *Impaired motor function in MPTP-induced PD mice*

**Figure 1:**
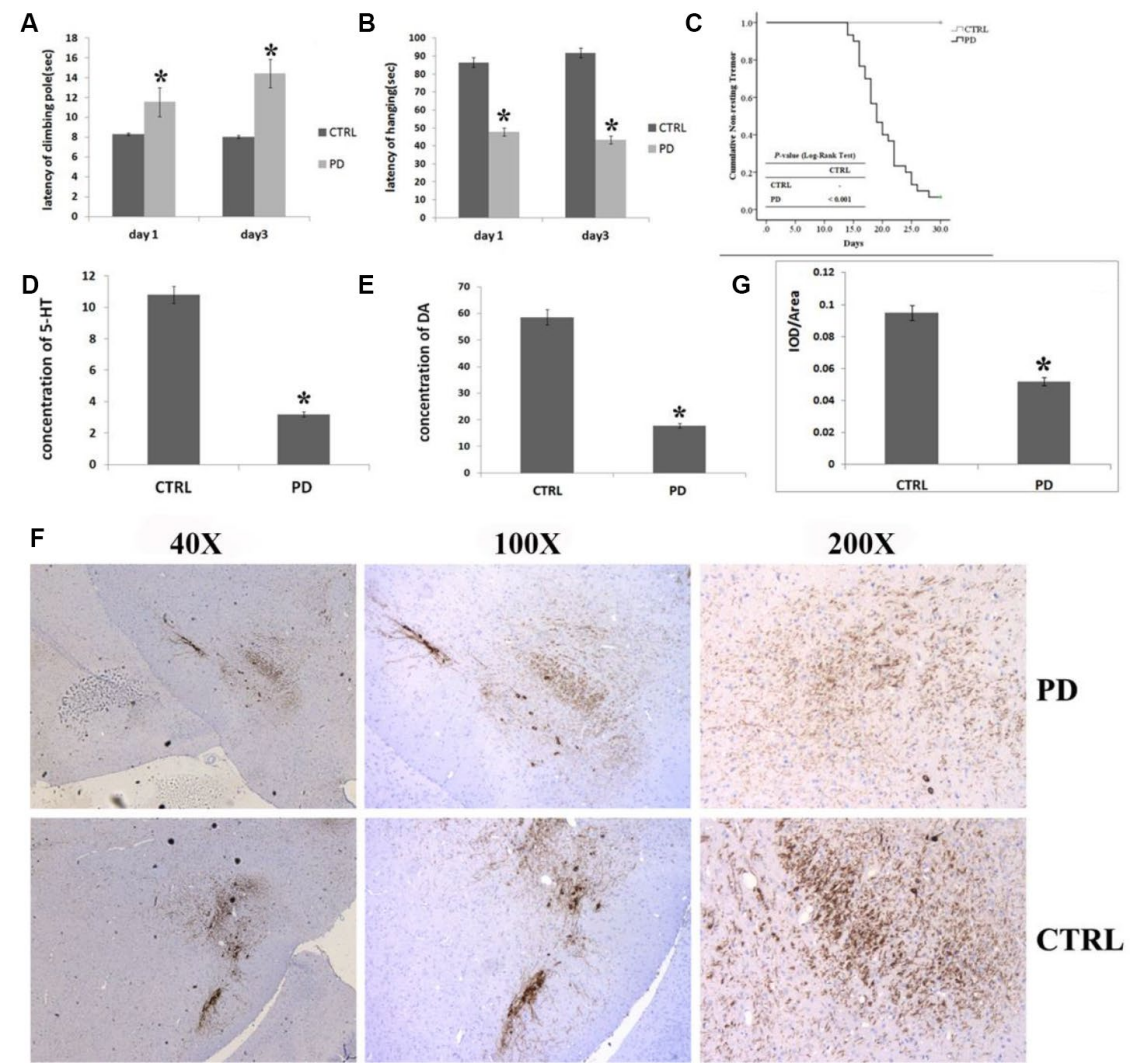
**Verification of PD in mice treated with MPTP.** (**A**–**C**) Effect of MPTP on motor function impairment by assess the time required to descend the pole in the vertical grid test (**A**), hanging duration in the horizontal grid test (**B**), and non-resting tremor using the Kaplan-Meier method and Log rank test (**C**). (**D**) Striatal 5-HT levels measured using HPLC. (**E**) Striatal DA levels measured using HPLC. (**F**) Representative photomicrographs of IHC staining for TH. (**G**) Statistical analysis of IHC staining for TH. In all panels, bars depict the mean±SD; *p<0.05.

The effect of MPTP on motor function was evaluated by comparing healthy and MPTP-induced mice. Figure 1 shows the significant behavioral impairment caused by MPTP. In vertical grid tests, MPTP-induced mice took significantly longer to descend a climbing pole (Day 1: 11.56±3.20 s, *p*<0.05; Day 3: 14.44±6.01 s, *p*<0.05) than control mice (Day 1: 8.31±2.58 sec, Day 3: 8.06±3.61sec) (Figure 1A). Moreover, the effect of MPTP appeared to be progressive, as latencies were significantly longer on day 3 than day 1. Similarly, in the horizontal grid test assess forepaw function, MPTP-induced mice were able to hang for significantly less time (Day 1: 47.69 ± 23.38 s, *p*< 0.05; Day 3: 43.31 ± 18.59 s, *p*< 0.05) than control mice (Day 1: 86.38 ± 39.40 s; Day 3: 91.77 ± 62.44 s), and again the effect of MPTP was progressive (Figure 1B).

MPTP-induced mice developed resting tremor on the 12^th^ day after the first injection, while control mice never developed a tremor. Kaplan-Meier analysis confirmed that MPTP-induced mice had a greater (*p*<0.01) tendency to develop resting tremor than control mice (Figure 1C).

### A significant reduction in striatal DA, 5-HT and in mesencephalic tyrosine hydroxylase (TH) was found in the MPTP-induced PD mice 

In support of the behavioral tests, we conducted an HPLC analysis of striatal DA and 5-HT levels and a histological analysis of TH in the substantia nigra par compacta (SNpc). MPTP-induced mice displayed a significantly lower striatal DA levels than control mice (PD: 17.82±10.06 μmol/L, CTRL: 58.57±8.73 μmol/L; *p*=0.006) (Figure 1E). Likewise, striatal 5-HT levels were significantly lower in MPTP-induced mice than control mice (PD: 3.19±2.10 μmol/L, CTRL: 10.80±3.21 μmol/L, *p*=0.012) (Figure 1D). Photomicrographs of IHC staining for TH in the SNpc showed that MPTP administration led to a massive loss of TH (Figure 1F). Quantification of the IHC results revealed that the mean staining intensity was reduced by 45.4% in MPTP-induced mice as compared to control mice (Figure 1G; the ratio of IOD to area, PD: 0.095±0.002; CTRL: 0.052±0.004, *p*<0.01).

### Enhanced tissue-specific aggregation of α-synuclein in the MPTP-induced PD mice

**Figure 2:**
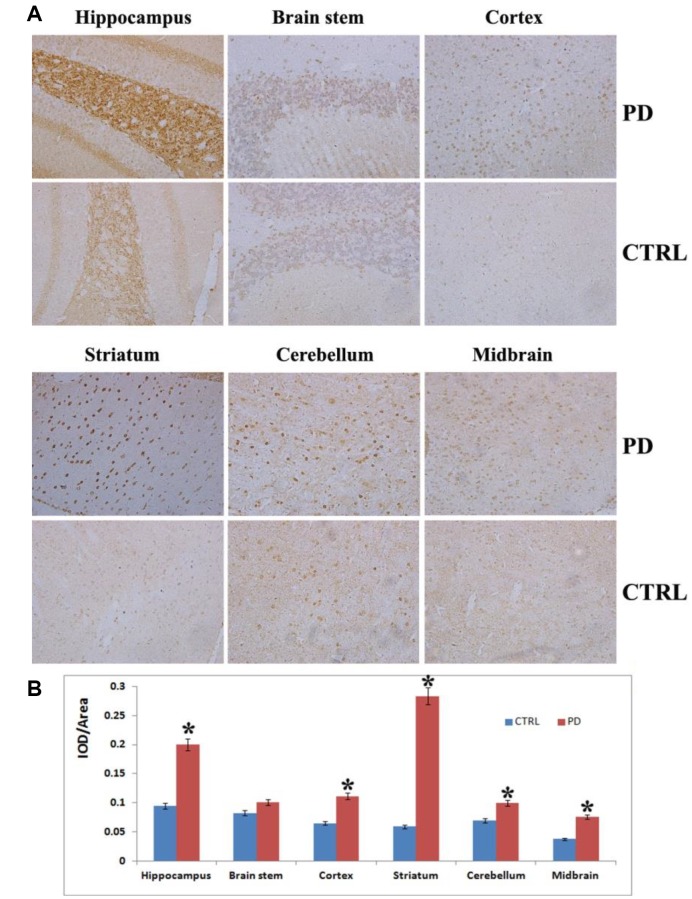
**Analysis on α-synuclein expression and aggregation in various brain areas.** (**A**) Representative images showing IHC staining for α-synuclein in the hippocampus, striatum, cortex, midbrain, cerebellum and brain stem (magnification: 100x). (**B**) Densitometric analysis of α-synuclein levels in the indicated brain areas. Bars depict the mean±SD; *p<0.05.

To further confirm the MPTP-induced PD mouse model, we assessed α-synuclein aggregation, a hallmark of PD, in various tissues brain areas. Photomicrographs of α-synuclein immunostaining showed α-synuclein aggregation in the hippocampus, cortex, striatum, cerebellum, midbrain and brain stem (Figure 2). The hippocampus showed greater (*p* < 0.05) α-synuclein immunoreactivity in MPTP-induced than control mice, but there was no clear α-synuclein aggregation in this tissue. By contrast, the striatum of MPTP-induced mice displayed significantly greater (*p* < 0.05) α-synuclein immunoreactivity as well as small, scattered, dot-like, dark-stained α-synuclein aggregates (Figure 2A). Strong α- synuclein immunoreactivity was also observed in the midbrain (*p* < 0.05), cortex (*p* < 0.05) and cerebellum (*p* < 0.05) in the MPTP-induced PD mice to along with weak aggregation of α-synuclein. No obvious α-synuclein immunoreactivity was detected in the brain stem of PD mice (*p*> 0.05) (Figure 2B). MPTP thus induces significant aggregation of α-synuclein in PD mouse brains, which damages DA nerve terminals and leads to loss of DA neurons from various brain regions. Taken together, these findings and the results of the motor function assessments indicate that MPTP-induced mice display the same characteristic Parkinsonian symptoms as human patients.

### Altered protein glycosylation in the brains of PD model mice

**Figure 3:**
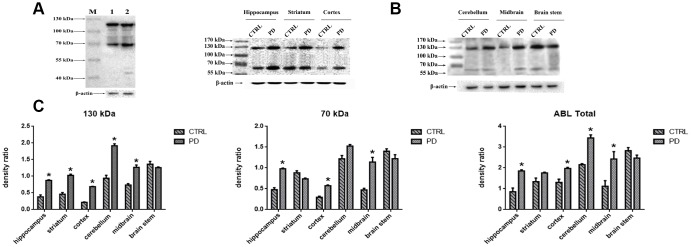
**Detection of altered protein glycosylation in various mouse brain areas.** (**A**) Western blot analysis of protein glycosylation in whole brain using ABL, which selectively labels Gal-(β-1,3)-GalNAc oligosaccharides: lane 1, control; lane 2, PD. (**B**) Western blot analysis of protein glycosylation in the indicated brain areas. (**C**) Densitometric analysis of Gal-(β-1,3)-GalNAc oligosaccharides levels normalized to the level of β-actin. Bars represented the mean±SD; * *p*<0.05 vs. control.

To evaluate the changes in protein glycosylation in MPTP-induced mice, we performed Western blot analyses using agaracus bisporus lectin (ABL), which specifically recognizes Gal-(β-1,3)-GalNAc oligosaccharides linked to glycoprotein. Proteins were labels in at least five bands at molecular weights ranging from 40 kDa to 130 kDa (Figure 3A). Of those, two major bands at approximately 70 kDa (Band-S) and 130 kDa (Band-L) were expressed at different levels in various brain areas (Figure 3B). To determine whether the altered protein glycosylation correlated with PD, the abundances of glycoproteins with exposed Gal-(β-1,3)-GalNAc oligosaccharides in selected areas of mouse brain were assessed relative to the levels of β-actin (Figure 3C). Densitometric analyses revealed significantly higher protein glycosylation in the midbrain (Band-L: +72.42%; *p*=0.030. Band-S: +141.50 %; *p*=0.005. Total: +117.06%; *p*=0.008), cortex (Band-L: +215.63%; *p*<0.001. Band-S: +94.52%; *p*=0.010. Total: +145.97%; *p*=0.02), striatum (Band-L: +113.28%; *p*<0.001. Band-S: -16.66%; *p*=0.057. Total: +31.37%; *p*=0.0140), hippocampus (Band-L: +130.52%; *p*<0.001. Band-S: +105.13%; *p*=0.010. Total: +116.36%; *p*<0.001), and cerebellum (Band-L: +104.31%; *p*=0.010. Band-S: +3.24%; *p*=0.260. Total: +85.20%; *p*<0.001) in the PD group than in the control group. On the other hand, no significant difference was detected in the brain stem (Band-L: -7.85%; *p*=0.297. Band-S: -12.78%; *p*=0.190. Total: -12.67%; *p*=0.056). It thus appears that protein glycosylation is altered in a region-specific manner in PD mice, and that two glycoproteins, in particular, are affected.

### ABL-labeled glycoprotein was identified as microtubule-associated protein 6 (MAP6)

**Figure 4:**
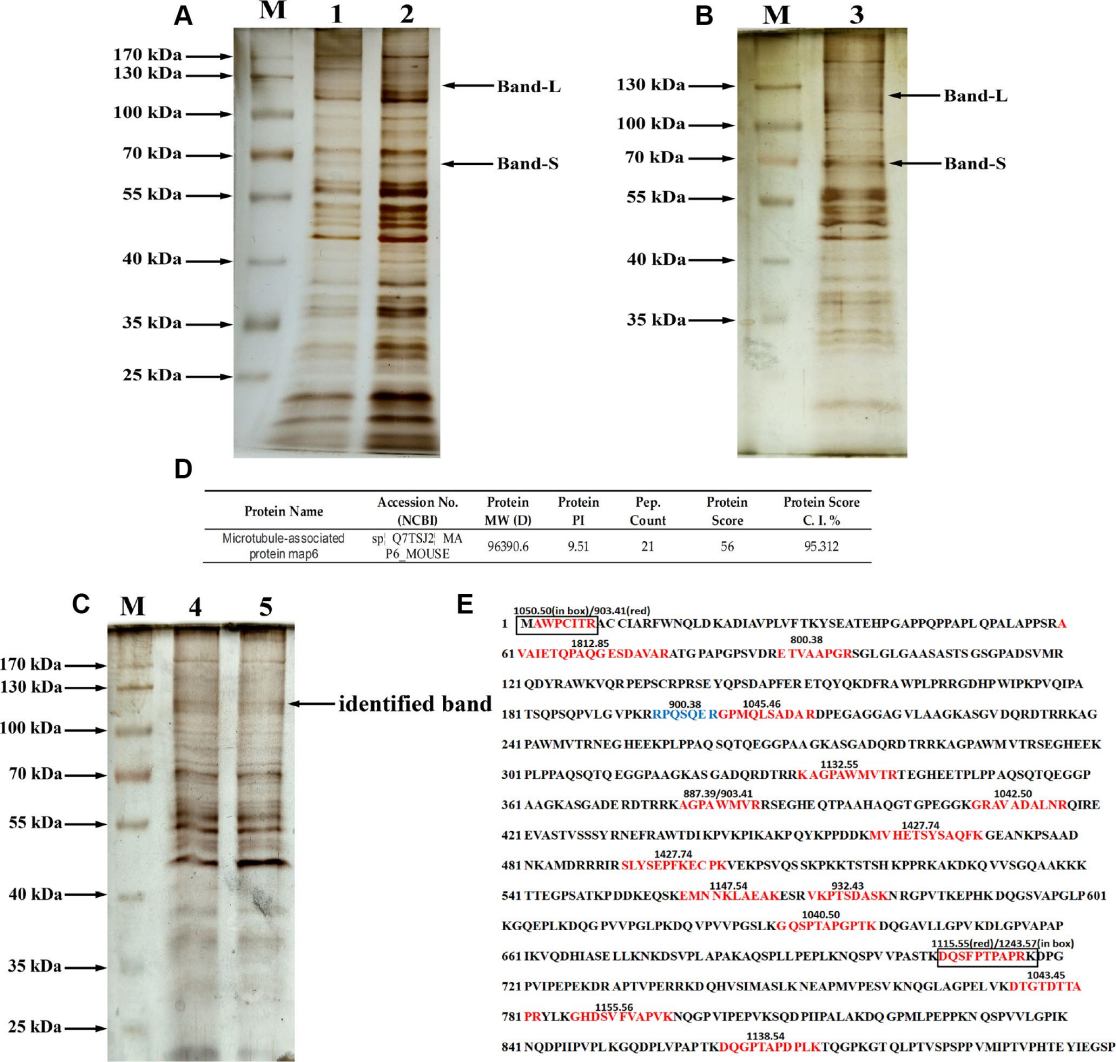
**Purification and identification of MAP6 via lectin affinity chromatography coupled with MALDI-TOF MS**. (**A**) SDS-PAGE analysis on the fractions from wash buffer containing 0.3 mol/L NaCl. Lanes 1 and 2 were the different fractions. Lane M, protein size standards. (**B**) SDS-PAGE analysis of the fractions from eluted buffer containing 0.5 mol/L NaCl. Lane 3, eluted fraction. Lane M, protein size standards. (**C**) SDS-PAGE analysis of purified glycoproteins and silver staining: lane M, protein size standards – the arrow shows the glycoprotein subjected to MS identification; Lanes 4 and 5, glycoproteins purified in lectin pulldown assays. (**D**) Profiles of identified MAP6 in Mus musculus. (**E**) Trypsin-digested MAP6 peptide fragments identified based on their MALDI-TOF MS spectrum. The identified peptide fragments are indicated in red, blue, and box. The numbers on the sequence are the m/z of the molecular fragment ions after collision-induced dissociation.

To identify the glycoproteins apparently affected by PD, lectin pull-down assays were coupled with tandem mass spectrometry. The purified glycoproteins were analyzed using 10% SDS-PAGE and silver staining as shown in Figure 4A–4C. At least 6 diverse glycoproteins were detected on the silver-stained gel at molecular weights ranging from 70 kDa to 170 kDa. In addition, compared to the non-specifically bound proteins (Figure 4A), mainly glycoproteins containing the Gal-(β-1,3)-GalNAc oligosaccharides had molecular weights more than 50 kDa, and the density of bands similar to Band-L and Band-S was greatest with the specifically bound glycoproteins (Figure 4B and 4C). Therefore, bands at molecular weights similar to those of Bands S and L detected by immunoblotting with ABL (the arrow in Figure 4C) were selected for MALDI-TOF mass spectrometric analysis. The MALDI-TOF mass spectrometry data with a protein score ≥ 50 at the 95% confidence level (protein score C.I.%) were used for identification of glycoproteins of interest. In this study, the MS analysis on glycoprotein of Band-L resulted in identification of microtubule-associated protein 6 (MAP6) as shown in Figure 4D.

The glycoprotein in Band-L yielded 19 fragment ions after collision-induced dissociation. The detected fragment ions m/z 800.3782, 887.3906, 900.3793, 903.4086, 932.425, 1034.4485, 1040.4956, 1042.4941, 1045.46, 1050.4974, 1115.5209, 1132.553, 1138.5381, 1147.5438, 1155.5581, 1243.5729, 1427.7357, 1649.77, 1765.8442, and 1812.8546 corresponded to the sequences indicated in Figure 4E. Sequence alignment analysis revealed that all of the identified fragment ions were consistent with trypsin-digested microtubule-associated protein 6 (MAP6). Additionally, the tandem mass spectrometry (MS/MS) spectrum confirmed that the *m/z* 1427.74 was composed of MVHETSYSAQFK, while *m/z* 903.409 was composed of AGPAWMVR, which only appeared in trypsin-digested MAP6. Based on MALDI-TOF spectral analysis, therefore, the glycoprotein in Band-L is strongly suspected to be MAP6.

### Confirmation of MAP6 glycosylation using in situ dual immunolabeling

**Figure 5:**
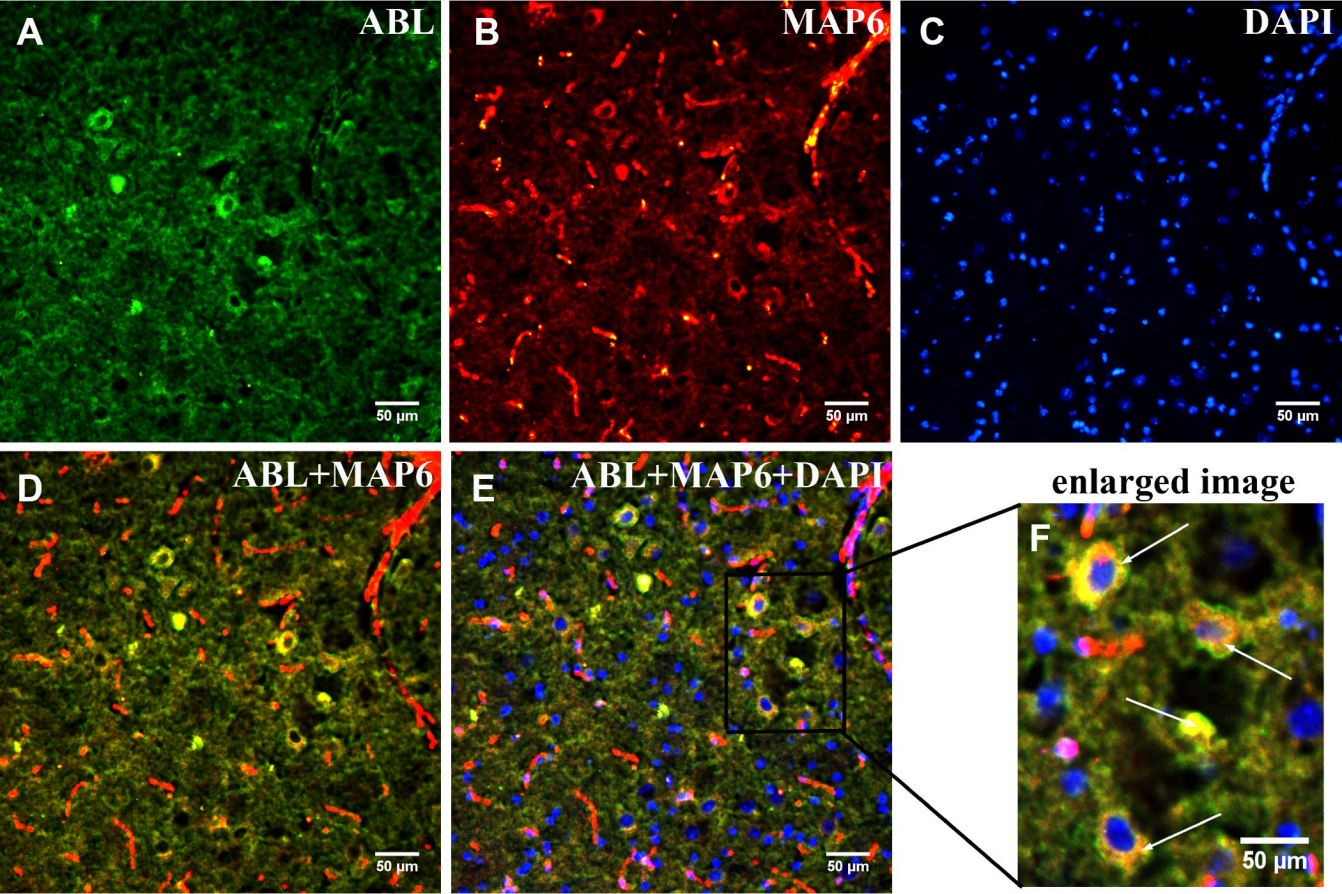
**Dual immunofluorescence analysis of MAP6 glycosylation in brainstems from MPTP-induced mice.** Photomicrographs show co-localization of MAP6 (**A**; red) and ABL (**B**; green). Nuclei are stained with DAPI (blue). Merged images show their colocalization (MAP6+ABL) (**C** and **D**; yellow). White arrows point to MAP6 with a glycoprotein. The enlarged image is the boxed part in the merged image.

To confirm that MAP6 was the glycoprotein in Band L, in situ dual co-immunofluorescence analysis of brainstems from MPTP-induced mice was carried out using a monoclonal anti-MAP6 antibody and biotinylated ABL. In Figure 5, the fluorescence signals in red (MAP6) or green (protein with exposed Gal-(β-1,3)-GalNAc oligosaccharides) show clear presence of both MAP6 (Figure 5A) and glycoprotein (Figure 5B) in PD mouse brainstem. Moreover, the merged images in Figure 5C and 5D reveal the co-localization of MAP6 with a glycosylated protein. These findings together with a the mass spectrometry data confirmed the glycoprotein of interest is MAP6.

### Confirmation of MAP6 as a glycoprotein using immunoprecipitation and WB analysis

**Figure 6:**
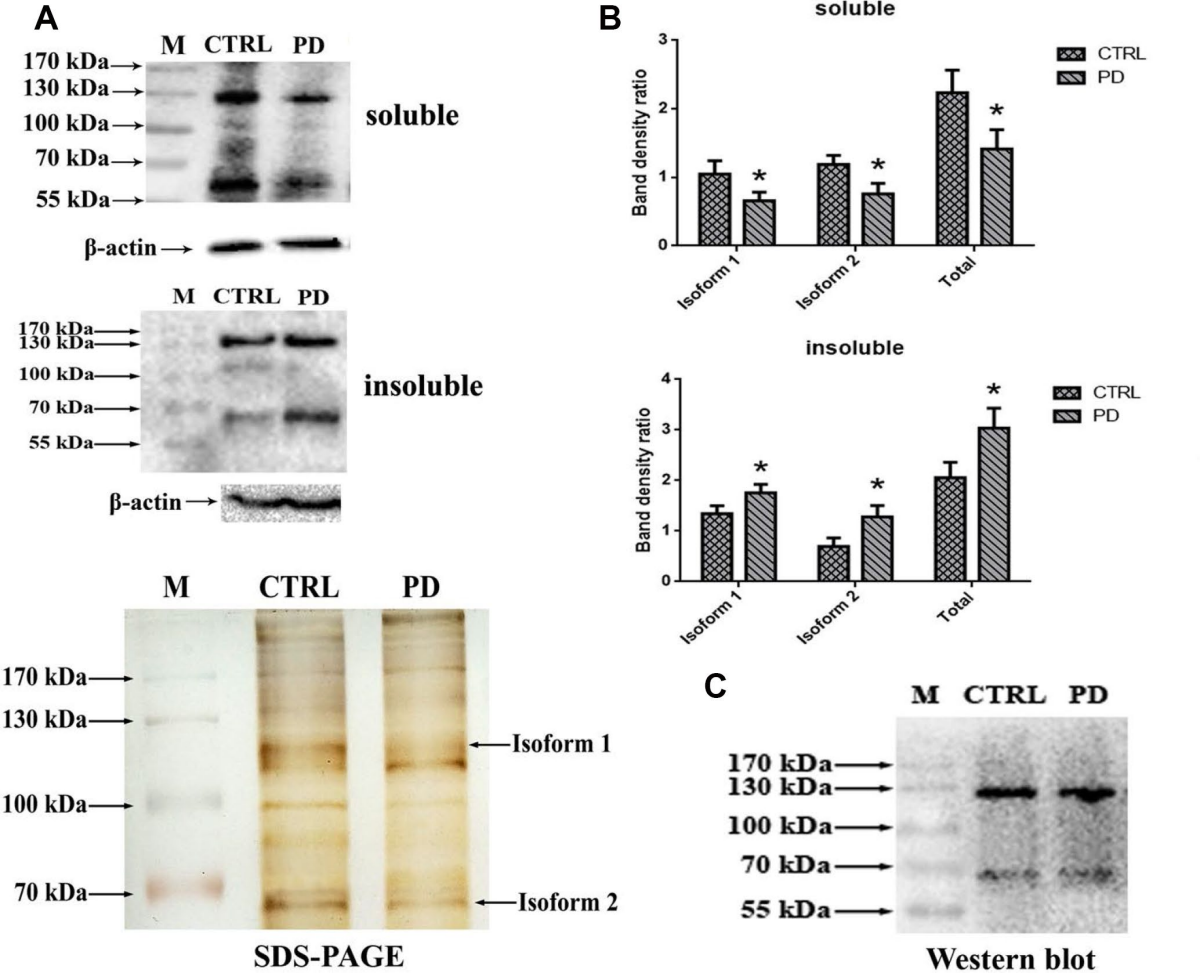
**Confirmation glycoprotein of interest is MAP6.** (**A**) Western blot analysis of soluble lysates and insoluble precipitates. M, protein size standards. (**B**) Densitometric analysis of MAP6 levels normalized to the level of β-actin in soluble lysates and insoluble precipitates. Bars depict the mean±SD; * *p*<0.05 vs. control. (**C**) Blots showing that MAP6 is a glycoprotein containing Gal-(β-1,3)-GalNAc oligosacchrides. Immunoprecipitation was coupled with Western blot analysis of extract from the whole mouse brain. The SDS-PAGE gel was visualized using silver-staining kit; and the membrane of WB was blotted with ABL, and visualized using a ECL kit.

To further test whether the identified glycoprotein of interest was MAP6, Western bolt analysis using monoclonal anti-MAP6 antibody as a probe was conducted. Proteins were separated through 10% SDS-PAGE, and the bands were visualized by ECL. Figure 6A shows that two clear bands appear on the membrane located at around 70 kDa and 130 kDa in hippocampus of mouse brains. Search of a public online database was used to obtain the information of MAP6 based on amino acid sequences. The sequence of MAP6 was retrieved from the following website: https://www.ncbi.nlm.nih.gov/protein/?term=microtubule-associated+protein+6+mouse. In mus musculus two isoforms of MAP6 were found, one is a 568-amino-acid protein with a predicted molecular weight of 61.11 kDa, and the other one is a 906-amino-acid protein with a predicted molecular weight of 96.45 kDa. The obtained sequences were aligned using the multiple sequence alignment program (Multalin). Based on the alignment analysis, it was found that the 568-amino-acid MAP6 (isoform 2) was the N-terminal fragment of the 906-amino-acid MAP6 (isoform 1). A statistical analysis revealed dramatically lower hippocampal levels of soluble MAP6 (Isoform 1: PD: 0.657±0.127; CTRL: 1.048±0.196; *p*=0.044. Isoform 2: PD: 0.759±0.155; CTRL: 1.188±0.134; *p*=0.022. Total: PD: 1.415±0.281; CTRL: 2.236±0.330; *p*=0.031) and higher levels of insoluble MAP6 (isoform 1: PD: 1.756±0.172; CTRL: 1.345±0.156; *p*=0.037. isoform 2: PD: 1.282±0.223; CTRL: 0.696±0.171; *p*=0.022. Total: PD: 3.038±0.395; CTRL: 2.040±0.327; *p*=0.027) was in tissue from MPTP-induced mice (Figure 6B). To further confirm that the MAP6 was glycosylated and carrying exposed Gal-(β-1,3)-GalNAc oligosacchrides, we purified MAP6 protein via immunoprecipitation using monoclonal anti-MAP6 antibody and then subjected the precipitated to Western blotting with ABL (Figure 6C). The images of the images of the blots and SDS-PAGE gels show the two strongest bands at the positions of around 70 kDa and 130 kDa, corresponding to molecular masses of the isoform1 and the isoform2 of MAP6, respectively. Taken together, these findings further confirm that MAP6 is a glycoprotein containing Gal-(β-1,3)-GalNAc oligosacchridese, and associated with PD pathogenesis via forming MAP6 precipitation.

### Glycosylated MAP6 is strongly associated with PD pathogenesis

**Figure 7:**
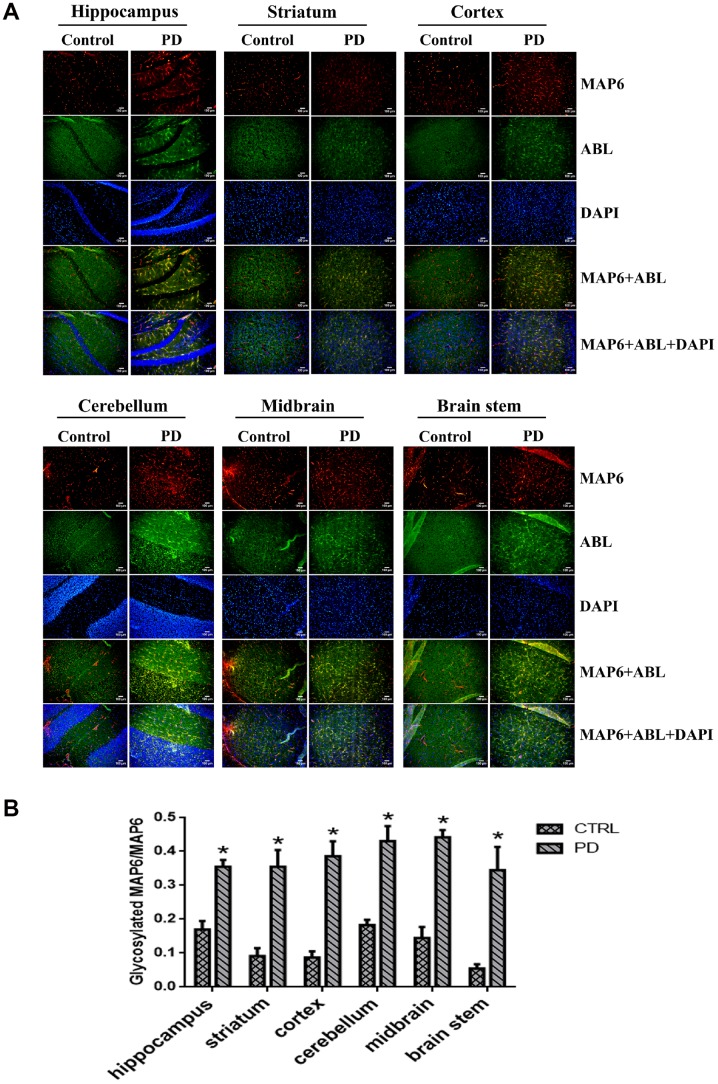
**In situ dual immunofluorescence analysis glycosylated MAP6 in various brain areas in PD mice**. (**A**) Representative photomicrographs of immunofluorescent labeling of glycoproteins detected by ABL (green) and MAP6 stained using anti-MAP6 monoclonal antibody (red) in the indicated areas, as well as their colocalization (yellow). Nuclei were stained using DAPI (blue). (**B**) Statistical comparison of the immunofluorescent signals between the MPTP-induced PD mice and control mice. Bars depict the mean±SD; *p<0.05 vs. control group.

Our results suggest glycosylation of MAP6 with Gal-(β-1,3)-GalNAc oligosaccharides correlates with PD in the MPTP-induced model. To determine whether MAP6 glycosylation is upregulated in PD, we carried out in situ double-immunofluorescent labeling of various mouse brain areas. Figure 7A shows the apparent expression and distributions of glycoproteins (green) and MAP6 (red) in the indicated areas, as well as their colocalization (yellow). Calculation of glycoMAP6-to-MAP6 ratios revealed that in MPTP-induced PD mice, glycosylated MAP6 levels were significantly elevated the hippocampus (PD: 0.355±0.034, CTRL: 0.170±0.042; *p*=0.004), cerebellum (PD: 0.430±0.079, CTRL: 0.183±0.027; *p*=0.007) and striatum (PD: 0.355±0.086, CTRL: 0.091±0.040; *p*=0.009), midbrain (PD: 0.441±0.039, CTRL: 0.145±0.055; *p*=0.002), brainstem (PD: 0.345±0.119, CTRL: 0.054±0.022; *p*=0.014), and cortex (PD: 0.386±0.076, CTRL: 0.087±0.031; *p*=0.003) (Figure 7B). Thus, widespread upregulation of MAP6 glycosylation appears to be associated with PD.

In addition, there was a marked difference in the cerebral MAP6 distribution between PD and control mice. In control mice, MAP6 was discretely distributed as dot-like structures evenly dispersed within of mouse brains. In PD mice, by contrast, MAP6 tended to accumulate into filamentary structures or larger agglomerates. We also found that MAP6 levels in the insoluble fraction of the hippocampus was higher in MPTP-induced mice than control mice (Figure 7C). These results suggest that glycosylation of MAP6 with Gal-(β-1,3)-GalNAc oligosaccharides may promote MAP6 redistribution or accumulation as insoluble inclusions within mouse brains.

## DISCUSSION

MAP6, also known as stable tubule only polypeptide (STOP), was initially characterized as a microtubule stabilizing protein within the MAP family of proteins. MAP6 has two major isoforms in vertebrates, which are products of alternatively spliced mRNAs or alternative promoters [20]. The major MAP6 isoforms in the mouse central nervous system are MAP6-E [21], which is expressed during neurodevelopment, and MAP6-N, which is expressed postnatally throughout the mouse brain and exhibits pronounced axonal localization [22–24]. Consistent with those studies, we identified two main MAP6 isoforms, which were widely distributed in various areas throughout the brain. Within the nervous system, microtubules are key components necessary for proper neuronal function, polarity and signaling. Microtubule stability depends largely on the function of several MAPs, including MAP1, MAP2, Tau and MAP6. MAPs regulate microtubule bundling, polymerization, and/or stabilization into neuronal microtubules arrays and, during brain development, contribute to a variety of neuronal functions involved in neuronal migration and polarization as well as axonal elongation and guidance [25–27]. Dysfunction of microtubules and/or MAPs often leads to psychiatric disorders and neurodegenerative diseases [28]. For example, MAP6 is currently being studied for its role in schizophrenia [25–27]. On the other hand, nothing is known about MAP6’s contribution to the pathogenesis of PD. Our results indicate that MAP6 is not detectable using ABL in the brains of healthy control mice, but is detected in the brains of MPTP-induced PD mice. This suggests that induction of PD is associated with the glycosylation of MAP6 with Gal-(β-1,3)-GalNAc oligosaccharides, which is consistent with the hypothesis that abnormal glycosylation is triggered by oxidative stress in PD [12]. Based on our observations in the present study, we further propose that oxidative stress in PD leads to MAP6 glycosylation with Gal-(β-1,3)-GalNAc oligosaccharide, which in turn enhances formation of insoluble inclusions. This is the first report of MAP6 glycosylated with Gal-(β-1,3)-GalNAc oligosaccharides. To better understand the composition and structure of the sugar chains linked to MAP6, we used NetNGlyc software to performed a predictive analysis based on the sequence of MAP6 in Mus musculus, the common house mouse. The 906-amino acid form of MAP6 was predicted to have no potential N-linked glycosylation sites, but to have a high potential for O-linked glycosylation clusters. ABL binds all O-linked glycans in IgA subclasses, which include three glycoforms: GalNAc-Ser/Thr, Gal-(β-1,3)-GalNAc-Ser/Thr, and GlcNAc-(β-1,4)-Gal-(β-1,3)-GalNAc-Ser/Thr. Under normal conditions, MAP6 carries O-linked glycans with the structure of oligosaccharide/monosaccharide-Gal-(β-1,3)-GalNAc-Ser/Thr, which is not recognized by ABL. But when MAP6 loses the terminal oligosaccharide/monosaccharide to expose the structure of Gal-(β-1,3)-GalNAc-Ser/Thr, as it apparently does after MPTP induction of PD, MAP6 can be detected by ABL. This scenario is consistent with the in situ dual co-immunofluorescence analysis in the present study, and with an earlier report indicating that maintenance of terminal monosaccharide/oligosaccharide levels may be a protective mechanism in neurodegenerative diseases [29]. However, further study will be needed to confirm the compositional and structural identification of the MAP6 oligosaccharide chain.

PD is characterized by degeneration of dopaminergic neurons, resulting in loss of the striatal neurotransmitter dopamine, which plays key roles in the modulation of movement, emotion, reward behavior, and motivation [30]. It is believed that the lack of dopaminergic innervation in the striatum is responsible for such Parkinsonian symptoms as bradykinesia, rigidity, resting tremor, and postural instability. Therefore, damage to the nigrostriatal dopaminergic pathway, which connects the SNpc with the striatum, is thought to be as a prominent feature of PD patients [31]. However, the precise functions of the nigrostriatal dopaminergic pathway are still not fully understood in healthy or PD states. In the present study, we investigated various brain areas in the nigrostriatal dopaminergic pathway, including the midbrain and striatum. Additionally, the hippocampus was investigated as a specific region affected by PD. Our results show that MAP6 glycosylated with Gal-(β-1,3)-GalNAc is significantly increased/accumulated within mouse brains affected by PD. We therefore hypothesize that in MPTP-induced PD model mice, MAP6 glycosylated with Gal-(β-1,3)-GalNAc may damage the neurons in the nigrostriatal dopaminergic pathway, ultimately disrupting dopaminergic neurotransmission. Consistent with that idea, we detected dramatically decreased striatal dopamine levels in these mice.

## MATERIALS AND METHODS

### Animal care and MPTP administration

This study was carried out in accordance with the principles of the Basel Declaration and recommendations of Dalian Medical University for laboratory animals. The protocol was approved by the Animal Ethics Committee of Dalian Medical University.

Thirty C57BL/6 male mice, aged 8 to 10 weeks and weighting 20-25 g, were randomly divided into control (n=15) and MPTP (n=15) groups. Mice in the MPTP group were administered intraperitoneal injections of MPTP (25 mg/kg or 10 ml/kg, dissolved in physiological saline) 10 times at intervals of 3.5 days, while control mice received the same volume of physiological saline (10 ml/kg).

### Neurobehavioral testing

The activities of the mice were observed and recorded each day. Motor dysfunction was assessed using horizontal and vertical grid tests, which were administered on the 1^st^ and 3^rd^ day after the last MPTP injection. In the horizontal grid test, horizontal locomotor activity was measured using a wire apparatus constructed as described previously [32]. The mouse was placed on the apparatus, facing upward. The period of time each mouse hung onto the wire was monitored in three independent trials with intervals of 20 min. In the vertical grid test, mice were placed head down on the top of a vertical wooden pole (50 cm in length and 1.5 cm in diameter) with a rough surface constructed as described previously [33]. The time required for each mouse to climb down the pole from the top to the base was measured as the average of three trails. Trials were considered a failure if the mouse slid down or jumped off the pole.

### Processing of brain tissues

On the 7^th^ day after the last MPTP or saline injection, eight of the 15 mice in each group were anesthetized by inhalation of diethyl ether. The chest was then opened to expose the heart, and the mice were perfused intracardially with 0.9% physiological saline and 4% paraformaldehyde in phosphate-buffered saline (PBS; 50 mmol/L of NaH_2_PO_4_; 5 mmol/L of KCl; 1.5 mmol/L of MgCl_2_; and 80.1 mmol/L of NaCl; pH 7.4) for 30 min each. From four of those mice in each group, the striatum, midbrain, brainstem, cortex, cerebellum, and hippocampus were carefully dissected, post-fixed in the same fixative, and stored at 4°C for immunohistochemical (IHC) examination. The whole brains of the other four mice in each group were saturated in 15% picric acid in PBS followed by storage in 20% sucrose in PBS at 4°C for immunofluorescent staining. The remaining seven mice in each group were sacrificed by cervical dislocation and washed with ice-cold 0.9% physiological saline. The hippocampus, striatum, brain stem, cortex, cerebellum, and midbrain were carefully dissected and frozen at –80 °C for a further investigation.

### Measurement of dopamine and 5-hydroxytrytamine by reverse-phase high performance liquid chromatography

Frozen tissues were homogenized in ice cold RIPA lysis buffer [50 mmol/L Tris (pH 7.4) containing 150 mmol/L NaCl, 1% Triton X-100, 1% sodium deoxycholate, 0.1% SDS and 1 mmol/L PMSF], and the soluble proteins were obtained via centrifugation (27,000*g*, 30 min, 4°C). Protein levels were then measured using a BCA kit (keyGEN BioTECH, China). After high abundance proteins were removed from the striatal supernatant by adding ice cold acetone, the levels of dopamine (DA) and 5-hydroxytryptamine (5-HT; serotonin) was measured with reverse-phase HPLC using a C18 reverse phase column (3.9 mm x 150 mm). The mobile phase was composed of buffer A (100% methanol) and buffer B (0.03% trifluoroacetic acid in Milli-Q water [V:V]). The C18 column was equilibrated with buffer A at a flow rate of 0.5 ml/min for 20 min, after which 20 μl of sample were injected, and a constant proportion of 7 to 93 (buffer A to buffer B) was delivered over a period of 60 min at the flow rate of 0.5 ml/min. The eluted compounds were detected by monitoring the UV signal at 280 nm. A standard curve was prepared using serial dilutions of DA and 5-HT and calculated using regression analysis of the peak areas.

### Western blot analysis

Aliquots (120 mg) of total proteins from each tissue were separated on 10% SDS-acrylamide gel electrophoresis (SDS-PAGE) and transferred to a PVDF membrane in blotting buffer (20 mmol/L Tris-base, 150 mmol/L glycine and 20 % methanol). The glycoprotein were then probed using lectin detection with biotinylated ABL (Vectorlabs), anti-β-actin monoclonal antibody (Abcam), and anti-microtubule-associated protein 6 monoclonal antibody (STOP (175): sc-53513, anti-MAP6, Santa Cruz Biotechnology, Santa Cruz, CA). Protein bands were visualized using an enhanced chemiluminescence system (GE Healthcare Bio-Sciences Corp.).

### Immunohistochemistry and immunofluorescent staining

For histological evaluation, fixed brain tissues were routinely processed for embedding in a paraffin followed by dehydration using an alcohol gradient (70%, 80%, 90%, 95%, 100%). The samples were then diaphanized by immersion in xylol and embedded in paraffin. The paraffin-embedded samples were cut into 10-μm-thick sections and mounted on glass slides for IHC analysis using anti-tyrosine hydroxylase (anti-TH, Santa Cruz Biotechnology, Santa Cruz, CA) and anti α-synuclein (Sigma) antibodies. Diaminobenzidine was used for visualization of the immunolabeling.

For immunofluorescent staining, whole mouse brains frozen in Milli-Q water cut into 8-μm-thick sections on a freezing microtome/cryostat (-40°C). The free floating sections were immunostained with biotinylated ABL, which specifically recognizes galactosyl (β-1,3) N-acetylgalactosamine [Gal-(β-1,3)-GalNAc] and anti-MAP6 monoclonal antibody. The immunolabeling was visualized using streptavidin-FITC and rhodamine-conjugated secondary antibody, respectively. The sections were counterstained with DAPI.

### Lectin pull-down assay and tandem mass spectrometric analysis

Twenty μl of Streptavidin Magnetic Agarose Beads (Invitrogen) were equilibrated in 200 μl of equilibration buffer (20 mmol/L, pH 8.0 Tris-HCl, 0.15 mol/L NaCl, 1 mmol/L MnCl_2_ and 1 mmol/L CaCl_2_). Thereafter, 3 μg of biotinylated ABL (bait) were added bead suspension, which was then rotated slowly for 1 h at 4°C. The supernatant was removed using magnetic MagRack6™, leaving only the magnetic beads with bound proteins. The bound biotinylated ABL was incubated with 200 μl soluble proteins (prey) from the whole-cell lysates of mouse brains for 1h at 4°C with gentle rotation. The beads were then washed with 200 μl washing buffer I (20 mmol/L, pH 8.0 Tris-HCl, 0.2 mol/L NaCl, 1 mmol/L MnCl_2_, 1 mmol/L CaCl_2_), and washing buffer II (20 mmol/L, pH 8.0 Tris-HCl, 0.3 mol/L NaCl, 1 mmol/L MnCl_2_, 1 mmol/L CaCl_2_) respectively to remove nonspecific proteins, after which the bound proteins were eluted with 50 μl of elution buffer (20 mmol/L, pH 8.0 Tris-HCl, 0.15 mol/L NaCl, 1 mmol/L MnCl_2_, 1 mmol/L CaCl_2_ and 200 mmol/L N-acetyl-galactosamine). Final protein concentrations in the eluate were determined using a BCA Protein Kit and analyzed on 7.5% SDS-PAGE. Colorimetric detection of protein bands was performed using a silver staining kit (Sigma). Silver-stained bands were excised and identified using tandem mass spectrometric analysis as described previously [34].

### Co-immunoprecipitation

Soluble lysates of whole mouse brains were prepared as described above. Co-immunoprecipitation was performed using a Capturem IP & Co-IP Kit (Takara Bio USA, Inc) as instructed by the manufacturer. The soluble proteins were pre-incubated with monoclonal anti-MAP6 antibody, then 500 μl pre-incubated mixture was transferred onto a spin column equilibrated with the provided Equilibration Buffer. After the unbound proteins were removed with the provided Wash Buffer, the bound protein was eluted with the provided Elution Buffer. Protein in the elute was quantified using a BCA kit.

### Image quantification and statistical analyses

Protein in bands were quantified densitometrically using Image J (version 1.42). The color images were converted to gray levels for quantification. Slight variations in background staining were corrected by subtracting background density. Digital analysis on the immunofluorescence graphs and immunohistochemistry images was performed using Image-Pro Plus software (version 6.0).

Statistical analyses were done using SPSS (version 19) and GraphPad Prism 6. Statistical differences between the PD and control (CTRL) groups were evaluated using a two-tailed, equal variance Student’s t-test. Behavioral data (hyperspasmia) were assessed using Kaplan-Meier analysis with time and cumulative non-resting tremor rate (CTRL, PD) as factors. Western blot and IHC data were analyzed in triplicate. Values of *p* <0.05 was considered statistically significant.

## CONCLUSIONS

In summary, the results of this study suggest that aberrant MAP6 glycosylation in the brains of MPTP-induced mice is strongly associated with the pathogenesis of their Parkinsonian symptoms. Despite the limitations of this study, this is the first report to demonstrate hyperglycosylation of MAP6 with exposed Gal-(β-1,3)-GalNAc oligosaccharides, or to reveal its potential role in the pathogenesis of PD. These findings provide potentially valuable information for developing new therapeutic targets for the treatment of PD as well as reliably prognostic biomarkers.
